# Two new species and a remarkable record of the genus *Dendronotus* from the North Pacific and Arctic oceans (Nudibranchia)

**DOI:** 10.3897/zookeys.630.10397

**Published:** 2016-11-09

**Authors:** Tatiana Korshunova, Nadezhda Sanamyan, Olga Zimina, Karin Fletcher, Alexander Martynov

**Affiliations:** 1 Koltzov Institute of Developmental Biology RAS, Vavilova Str. 26, 119334 Moscow, Russia; 2 Branch of Pacific Geographical Institute FEB RAS, Partizanskaya Str. 6, 683000 Petropavlovsk-Kamchatsky Russia; 3 Murmansk Marine Biological Institute, Vladimirskaya Str.17, 183010 Murmansk, Russia; 4 Port Orchard 98366, Washington, USA; 5 Zoological Museum, Moscow State University, Bolshaya Nikitskaya Str. 6, 125009 Moscow, Russia

**Keywords:** Arctic Ocean, Dendronotus, molecular phylogeny, new species, North Pacific Ocean, Nudibranchia, taxonomy

## Abstract

Two new species of the nudibranch genus *Dendronotus*, *Dendronotus arcticus* **sp. n.** and *Dendronotus robilliardi* **sp. n.**, are described from the Arctic and North Pacific oceans respectively, based on morphological and molecular data, and the North Pacific *Dendronotus albus* is revealed to be a species complex. The species *Dendronotus robilliardi* **sp. n.** is described from the northwestern Pacific (Kamchatka) differing from the northeastern Pacific *Dendronotus albus* by molecular and morphological data. The synonymy of *Dendronotus diversicolor* with *Dendronotus albus* is confirmed by analysis of their original descriptions. An endemic Arctic species *Dendronotus arcticus* **sp. n.** is also described here, differing substantially from all species of the genus *Dendronotus* using morphological and molecular data. An unusual record of the recently described *Dendronotus kamchaticus* Ekimova, Korshunova, Schepetov, Neretina, Sanamyan, Martynov, 2015 is also presented, the first from the northeastern Pacific, geographically separated from the type locality of this species in the northwestern Pacific by a distance *ca.* 6000 km; molecular data show them to belong to the same species.

## Introduction

The species of the genus *Dendronotus* are common marine invertebrates of the shallow waters in the northern hemisphere. Gordon Robilliard presented a detailed review of the genus *Dendronotus* (Robilliard 1970), and noticed an unequal number of *Dendronotus* species in the major marine regions of the northern hemisphere. In the North Atlantic at that time only two species were known, whereas in the North Pacific seven species had been recorded. Robilliard commented that “This may be an actual biological phenomenon with the northeast Pacific being the centre of evolution and radiation of the genus but it is more likely a reflection of the collection pressure” (Robilliard 1970: 475). Further studies have confirmed this prognosis, but some unexpected patterns were also revealed.

Mikael Thollesson challenged a long-standing view that only a single polymorphic species *Dendronotus frondosus* (Ascanius, 1774) inhabits European waters (Thollesson 1998). Based on morphological features and the application of allozyme electrophoresis he showed the valid status of *Dendronotus lacteus* (Thompson, 1840), which had been omitted from faunal lists for more than one century (e.g. Odhner 1907, Thompson and Brown 1984, Roginskaya 1987). Later, the validity of *Dendronotus lacteus* was confirmed in a first molecular phylogenetic study of the genus *Dendronotus* based on the 16S gene (Stout et al. 2010). They also found that one more traditional synonym of *Dendronotus frondosus* was also a valid taxon, *Dendronotus venustus* MacFarland, 1966 from the northeastern Pacific. It was originally described by Frank Mace MacFarland in his famous volume on the North American opisthobranchs (MacFarland 1966).

Stout et al. (2010: 7) also suggested that “Further examination of additional specimens from various locations may reveal a complex of species currently considered to be *Dendronotus frondosus*.” This was fulfilled in a recent revision of the genus *Dendronotus* by Ekimova et al. (2015). In this study two new species (*Dendronotus kalikal* Ekimova, Korshunova, Schepetov, Neretina, Sanamyan & Martynov, 2015 and *Dendronotus kamchaticus* Ekimova, Korshunova, Schepetov, Neretina, Sanamyan & Martynov, 2015) were described from the northwestern Pacific Kamchatka waters, both of which are very similar externally to *Dendronotus frondosus*. A further species, *Dendronotus primorjensis* Martynov, Sanamyan, Korshunova, 2015, was described from the Sea of Japan (Martynov et al. 2015a, b; Korshunova et al. 2016). Thus, *Dendronotus frondosus* is shown to be a species complex.

To date, the majority of new cryptic/semi-cryptic species of the genus *Dendronotus* from the North Pacific were discovered mostly in the *Dendronotus frondosus* species complex while other species from the North Pacific do not appear to contain obvious cryptic species complexes. Two long-debated supposedly cryptic species, *Dendronotus albus* MacFarland, 1966 and *Dendronotus diversicolor* Robilliard, 1970, were recently synonymised by Stout et al. (2010) and, while this paper was in review, *Dendronotus diversicolor* was removed from synonymy with *Dendronotus albus* by Ekimova et al. (prepublication).

*Dendronotus albus* and *Dendronotus diversicolor* are difficult to distinguish morphologically (Robilliard 1970; Behrens 1980, 2006) and show no genetic differences (Stout et al. 2010). Surprisingly, a high rate of the genetic divergence between supposed *Dendronotus albus* from the NW Pacific Kamchatka region (Martynov et al. 2015b) and real *Dendronotus albus* (= *Dendronotus diversicolor*, see Discussion) from the NE Pacific was found in the present study. The molecular differences were confirmed from four variably coloured specimens and an egg mass from the NW Pacific. Therefore a semi-cryptic species from the *Dendronotus albus* species complex is discovered for the first time from NW Pacific and is described here as a new species, *Dendronotus robilliardi* sp. n., in recognition of the pioneering work of Gordon Robilliard.

The second new species originates from one of the coldest region of the world, the Laptev Sea in the eastern Arctic Ocean. According to morphological and molecular data, this species differs substantially from all known *Dendronotus* species, and is described in this work as *Dendronotus arcticus* sp. n.

Finally, a remarkable new record of the recently described *Dendronotus kamchaticus* is also presented, from the NE Pacific (Washington State, USA); it is separated from the type locality in the NW Pacific (Kamchatka) by a distance *ca.* 6000 km.

## Material and methods

### Collecting data

Four specimens of *Dendronotus arcticus* sp. n. were collected in the Arctic Laptev Sea by trawling by Olga Zimina. Four specimens and one egg mass of *Dendronotus robilliardi* sp. n. were collected in the NW Pacific, Kamchatka by SCUBA diving by Nadezhda Sanamyan. A single specimen of *Dendronotus kamchaticus* and a single specimen of *Dendronotus albus* were collected in the NE Pacific, Washington State, by SCUBA diving by Karin Fletcher. For molecular phylogenetic analysis single specimens of *Doto coronata* and *Tritonia plebeia* were collected in the Barents Sea (Dalne-Zelenetskaya Bay) and Norway (Gulen Dive Resort), respectively, by SCUBA diving by Tatiana Korshunova and Alexander Martynov. All specimens were preserved in 80–95% EtOH.

### Morphological analysis

All specimens were examined with a stereomicroscope (MBS-9) and photographed using digital cameras (Nikon D-90 and D-810) with a set of extension rings. The pharynges were removed and processed with a weak solution of domestic bleach (NaOCl). Jaws were examined using a stereomicroscope and digital cameras. The radulae were examined under a scanning electron microscope (CamScan Series II) at the electron microscopy laboratory of the Biological Faculty of Moscow State University.

### Molecular analysis

A total of eleven specimens and one egg mass was successfully sequenced for the mitochondrial genes cytochrome c oxidase subunit I (COI) and 16S, and also the nuclear gene 28S (C1-C2 domain). Additional sequences including outgroup specimens were obtained from GenBank (see Table 1 for full list of samples, localities, and voucher references).

**Table 1.** List of specimens used for phylogenetic analyses. New specimens are highlighted in bold.

Species	Voucher	Locality	GenBank accession nos.		
			COI	16S	28S
***Dendronotus albus* MacFarland, 1966** (= *Dendronotus diversicolor* Robilliard, 1970)	**ZMMU:Op-566**	**USA: Washington**	**KX788135**	**KX788123**	**KX788114**
*Dendronotus albus* MacFarland, 1966 (= *Dendronotus diversicolor* Robilliard, 1970)	LACM:174845	USA: California	-	GU339185	-
*Dendronotus albus* MacFarland, 1966 (= *Dendronotus diversicolor* Robilliard, 1970)	LACM:174846	USA: California	-	GU339186	-
***Dendronotus arcticus* sp. n.**	**ZMMU:Op-561**	**Russia: Laptev Sea**	**KX788140**	**KX788129**	**KX788118**
***Dendronotus arcticus* sp. n.**	**ZMMU:Op-562**	**Russia: Laptev Sea**	**KX788141**	**KX788130**	**KX788119**
***Dendronotus arcticus* sp. n.**	**ZMMU:Op-563**	**Russia: Laptev Sea**	**KX788142**	**KX788131**	**KX788120**
*Dendronotus dalli* Bergh, 1879	ZMMU:Op-295	Russia: Kamchatka	KM397001	KM397083	KM397042
*Dendronotus dalli* Bergh, 1879	ZMMU:Op-330	Russia: Kamchatka	KM396999	KM397081	KM397040
*Dendronotus dalli* Bergh, 1879	ZMMU:Op-331	Russia: Kamchatka	KM397000	KM397082	KM397041
*Dendronotus frondosus* (Ascanius, 1774)	ZMMU:Op-324	Russia: Barents sea	KM396980	KM397062	KM397021
*Dendronotus frondosus* (Ascanius, 1774)	ZMMU:Op-359	Russia: Barents sea	KM396979	KM397061	KM397020
*Dendronotus frondosus* (Ascanius, 1774)	ZMMU:Op-380	Norway	KM396976	KM397056	KM397017
*Dendronotus frondosus* (Ascanius, 1774)	ZMMU:Op-382	Russia: Barents sea	KM396977	KM397050	KM397018
*Dendronotus kamchaticus* Ekimova et al., 2015	ZMMU:Op-246.2	Russia: Kamchatka	KM396989	KM397072	KM397030
*Dendronotus kamchaticus* Ekimova et al., 2015	ZMMU:Op-247.1	Russia: Kamchatka	KM396991	KM397073	KM397032
*Dendronotus kamchaticus* Ekimova et al., 2015	ZMMU:Op-247.2	Russia: Kamchatka	KM396992	KM397074	KM397033
***Dendronotus kamchaticus*Ekimova et al., 2015**	**ZMMU:Op-565**	**USA: Washington**	**KX788144**	**KX788111**	**KX788121**
*Dendronotus kalikal* Ekimova et al., 2015	ZMMU:Op-284.3	Russia: Kamchatka	KM396988	KM397070	KM397029
*Dendronotus lacteus* (W. Thompson, 1840)	ZMMU:Op-288	Russia: Barents Sea	KM396975	KM397059	KM397016
*Dendronotus lacteus* (W. Thompson, 1840)	ZMMU:Op-335	Russia: Barents Sea	KM396973	KM397057	KM397014
*Dendronotus lacteus* (W. Thompson, 1840)	ZMMU:Op-383.1	Norway	KM396971	KM397054	KM397012
*Dendronotus niveus* Ekimova et al., 2015	ZMMU:Op-269	Russia: White Sea	KM396996	KM397078	KM397037
*Dendronotus niveus* Ekimova et al., 2015	ZMMU:Op-274.2	Russia: Barents Sea	KM396993	KM397076	KM397034
*Dendronotus niveus* Ekimova et al., 2015	ZMMU:Op-279	Russia: Barents Sea	KM396995	KM397077	KM397036
*Dendronotus patricki* Stout et al., 2011	SIO-BIC M12133	USA: California	HQ225828	HQ225829	-
*Dendronotus primorjensis* Martynov et al., 2015	ZMMU:Op-419	Russia: Japan Sea	KX672010	KX672008	KX672006
*Dendronotus primorjensis* Martynov et al., 2015	ZMMU:Op-420	Russia: Japan Sea	KX672011	KX672009	KX672007
*Dendronotus regius* Pola & Stout, 2008	CASIZ179492	Philippines	HM162708	HM162629	-
*Dendronotus regius* Pola & Stout, 2008	CASIZ179493	Philippines	JN869451	JN869407	-
***Dendronotus robilliardi* sp. n.**	**ZMMU:Op-567**	**Russia: Kamchatka**	**KX788136**	**KX788124**	**KX788115**
***Dendronotus robilliardi* sp. n.**	**ZMMU:Op-568**	**Russia: Kamchatka**	**KX788138**	**KX788126**	**KX788116**
***Dendronotus robilliardi* sp. n.**	**ZMMU:Op-569**	**Russia: Kamchatka**	**KX788137**	**KX788125**	**KX788112**
***Dendronotus robilliardi* sp. n.**	**ZMMU:Op-447**	**Russia: Kamchatka**	**KX788139**	**KX788127**	**KX788117**
***Dendronotus robilliardi* sp. n. egg mass**	**ZMMU:Op-570**	**Russia: Kamchatka**	**KX788143**	**KX788128**	-
*Dendronotus robustus* Verrill, 1870	ZMMU:Op-343	Russia: Barents sea	KM397002	KM397084	KM397043
*Dendronotus robustus* Verrill, 1870	ZMMU:Op-344	Russia: Barents sea	KM397003	KM397085	KM397044
*Dendronotus robustus* Verrill, 1870	ZMMU:Op-390.5	Russia: Barents sea	KM396968	KM397051	KM397009
*Dendronotus venustus* MacFarland, 1966	LACM174850	USA: California	HM162709	HM162630	-
*Dendronotus venustus* MacFarland, 1966	LACM:174852.1	USA: California	-	GU339199	-
***Doto coronata* (Gmelin, 1791)**	**ZMMU:Op-571**	**Russia: Barents sea**	**KX788145**	**KX788133**	**KX788113**
*Doto koenneckeri* Lemche, 1976	CASIZ178247	Portugal: Azores Islands	HM162735	HM162658	-
*Marionia arborescens* Vayssiere, 1877	CAS:177735	Philippines	KP226855	KP226859	-
*Notobryon thompsoni* Pola et al., 2012	CASIZ176362	South Africa	JN869456	JN869413	-
*Notobryon wardi* Odhner, 1936	CASIZ177540	Philippines	JN869454	JN869411	-
***Tritonia plebeia* Johnston, 1828**	**ZMMU:Op-572**	**Norway**	**KX788134**	**KX788122**	**KX788132**

Small pieces of tissue were used for DNA extraction with Diatom™ DNA Prep 100 kit by Isogene Lab, according to the producer’s protocols. Extracted DNA was used as a template for the amplification of partial sequences of the COI, 16S, and 28S. The primers that were used for amplification are LCO 1490 (GGTCAACAAATCATAAAGATATTGG, Folmer et al. 1994); HCO 2198 (TAAACTTCAGGGTGACCAAAAAATCA, Folmer et al. 1994); 16S arL (CGCCTGTTTAACAAAAACAT, Palumbi et al. 2002); 16S R (CCGRTYTGAACTCAGCTCACG, Puslednik and Serb 2008); 28S C1' (ACCCGCTGAATTTAAGCAT, Dayrat et al. 2001); and 28S C2 (TGAACTCTCTCTTCAAAGTTCTTTTC, Le et al. 1993). Polymerase chain reaction (PCR) amplifications were carried out in a 20-µL reaction volume, which included 4 µL of 5x Screen Mix (Eurogen Lab), 0.5 µL of each primer (10 µM stock), 1 µL of genomic DNA, and 14 µL of sterile water. The amplification of COI and *28S* was performed with an initial denaturation for 1 min at 95°C, followed by 35 cycles of 15 sec at 95°C (denaturation), 15 sec at 45°C (annealing temperature), and 30 sec at 72°C, with a final extension of 7 min at 72 °C. The 16S amplification began with an initial denaturation for 1 min at 95°C, followed by 40 cycles of 15 sec at 95°C (denaturation), 15 sec at 52°C (annealing temperature), and 30 sec at 72°C, with a final extension of 7 min at 72°C. Sequencing for both strands proceeded with the ABI PRISM® BigDye™ Terminator v. 3.1. Sequencing reactions were analysed using an Applied Biosystems 3730 DNA Analyzer. Protein-coding sequences were translated into amino acids for confirmation of the alignment. All sequences were deposited in GenBank (Table 1, highlighted in bold).

Original data and publicly available sequences were aligned with the MUSCLE algorithm (Edgar 2004). Separate analyses were conducted for COI (641 bp), 16S (462 bp), and 28S (350 bp). An additional analysis was performed with all three concatenated markers (1453 bp). Evolutionary models for each data set were selected using MrModelTest 2.3 (Nylander et al. 2004) under the Akaike information criterion (Akaike 1974). The HKY+I+G model was chosen for COI. The GTR + I + G model was chosen for 16S and for the combined dataset. The GTR+G model was chosen for 28S. Two different phylogenetic methods, Bayesian inference (BI) and Maximum likelihood (ML) were used to infer evolutionary relationships. Bayesian estimation of posterior probability was performed in MrBayes 3.2. Markov chains were sampled at intervals of 500 generations. Analysis was started with random starting trees and 10^7^generations. Maximum likelihood-based phylogeny inference was performed in GARLI 2.0 (Zwickl 2006) with bootstrap in 1000 pseudo-replications. The program TRACER v1.6 was used to examine the convergence results. Final phylogenetic tree images were rendered in the FigTree 1.4.2. The ABGD program is available from http://wwwabi.snv.jussieu.fr/public/abgd/abgdweb.html. COI and 16S FASTA alignments were analysed separately (excluding outgroups) using both proposed models Jukes-Cantor (JC69) and Kimura (K80). The program Mega7 (Kumar et al. 2016) was used to calculate the uncorrected p-distances between all the sequences. Pairwise uncorrected p-distances within and between clades were also examined.

## Results

### Phylogenetic analysis

In this molecular study, 44 specimens and one egg mass were included, representing 20 species and 120 sequences. The resulting combined tree provided better resolution than COI, 16S, or 28S separately (not shown). Trees of both Bayesian Inference (BI) and Maximum Likelihood (ML) were used to infer phylogenetic trees. The combined dataset yielded a sequence alignment of 1453 positions. The topology of the tree obtained by ML was the same as the one inferred by BI.

The molecular phylogenetic analysis (Fig. 5) support the presence of two distinct species, *Dendronotus arcticus* sp. n. (PP = 1, BS = 100%) and *Dendronotus robilliardi* sp. n. (PP = 1, BS = 99%). All *Dendronotus albus* specimens cluster together with maximum support (PP = 1, BS = 100%) and form a separate clade. The *Dendronotus kamchaticus* specimens from Kamchatka and from the NE Pacific (Washington State) cluster together in a single clade with maximum support (PP = 1, BS = 100%). The *Dendronotus kalikal* specimens are also clustered in a single clade but hold an unstable position on the tree (and are therefore excluded from further phylogenetic analysis). This could be explained because some of *Dendronotus kalikal* sequences are too short.

The ABGD analysis revealed fourteen potential genetic groups both for COI (the prior maximal distance ranged between 0.001 and 0.013) and 16S (the prior maximal distance ranged between 0.001 and 0.02) genes: *Dendronotus regius*, *Dendronotus robilliardi* sp. n., *Dendronotus arcticus* sp. n., *Dendronotus lacteus*, *Dendronotus kamchaticus* (including the specimen from USA), *Dendronotus niveus*, *Dendronotus dalli*, *Dendronotus albus*, *Dendronotus venustus*, *Dendronotus primorjensis*, *Dendronotus frondosus*, *Dendronotus patricki*, *Dendronotus robustus*, and *Dendronotus kalikal*.

The sensitivity of the species delineation methods are discussed in e.g. Jörger et al. (2012), Jörger and Schrödl (2013), Padula et al. (2014). To define species, we use an integrative approach (Dayrat 2005) including tree topologies, pairwise uncorrected distances, and ABGD as well as morphological data.

### Taxonomy Family Dendronotidae

#### Dendronotus arcticus sp. n.

Taxon classificationAnimaliaNudibranchiaDendronotidae

http://zoobank.org/6B4A9064-A864-498C-BC81-C2A00FBB6186

Figs 1 , 3A

##### Type material.

Holotype, ZMMU Op-561, 19 mm long (preserved), Laptev Sea, R/V “Dalnie Zelentsy”, sta. O-48, 74°34.9'N–74°35.1'N, 115°43.4'E–115°42.2'E, 04.10.2014, depth 15 m, drague, sand, collector O.L. Zimina. 3 paratypes, ZMMU Op-562–Op-564, same locality and collectors as holotype.

##### Type locality.

Laptev Sea.

##### Etymology.

After the Arctic region.

##### Diagnosis.

5–6 pairs dorsolateral appendages, colour brownish with scattered distinct opaque white dots, central tooth with up to 14 small denticles and reduced furrows, vas deferens moderate in length, penis long, bent.

##### Description.

**Figure 1.**
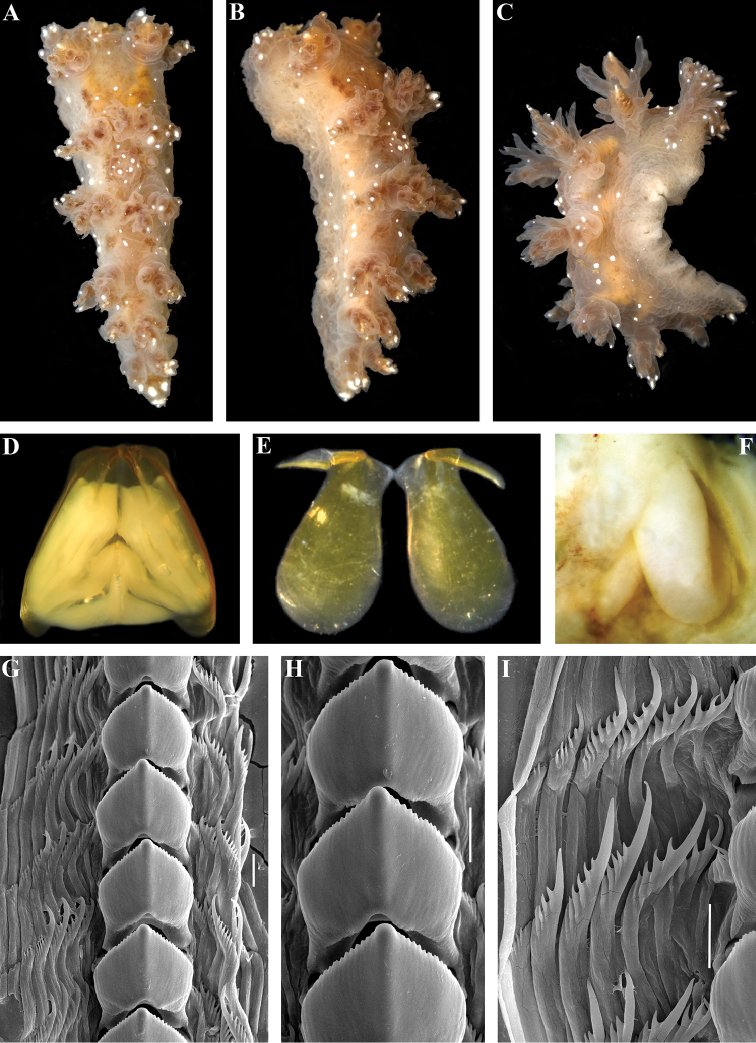
*Dendronotus arcticus* sp. n.: **A** holotype ZMMU Op-561, live, dorsal view **B** same, lateral view **C** paratype ZMMU Op-562, live, lateral view **D** holotype ZMMU Op-561, jaws and radula *in situ*, dorsal view **E** same, jaws, lateral views **F** same, penis **G** same, posterior rows of radula, SEM **H** same, details of central teeth, SEM **I** same, details of lateral teeth, SEM. Scale bars 30 µm. Photos of living specimens by Olga Zimina, other photos and SEM images by Alexander Martynov.

Body elongate, up to 19 mm in length (Fig. 1A–C), 6–8 branched appendages of oral veil, 5–6 appendages of rhinophoral stalks, 15–18 rhinophoral lamellae, branched rhinophoral lateral papilla present, 5–6 pairs dorsolateral appendages, 15–25 lip papillae. Dorsolateral appendages with moderate primary stalk, moderately branched secondary branches, and elongated tertiary branches (Fig. 1A–C). Reproductive and anal openings placed laterally on right side. General colour brownish with scattered distinct opaque white dots on notum, tips of lateral appendages, oral appendages, lip papillae, and rhinophores (Fig. 1A–C).

Dorsal processes of jaws inclined posteriorly at approximately 55° to longitudinal axis of jaw body and 0.47 of its length (Fig. 1D, E). Masticatory processes apparently bear indistinct denticles. Radula formula is 38 × 3–9.1.9–3. Central tooth weakly denticulated and bearing up to 14 small denticles (Fig. 1 G, H) with reduced furrows. Lateral teeth are short, slightly curved, bearing up to nine long denticles (Fig. 1I).

Reproductive system triaulic (Fig. 3A), ampulla twice folded, prostate consisting of 25–30 alveolar glands, vas deferens moderate in length expanding to voluminous penial sheath, vagina long and twisted, penis long and twisted (Fig. 1F), and bursa copulatrix is large, rounded, and elongated with small seminal receptaculum placed distally (Fig. 3A) (nomenclature of the seminal reservoirs according to Stout et al. 2011).

##### Biology.

Inhabits soft substrates (sand, mud) with gravel and small stones.

##### Distribution.

Central and eastern coastal waters of Arctic Ocean.

##### Remarks.

*Dendronotus arcticus* sp. n. is well separated from other species of the genus *Dendronotus*: externally *Dendronotus arcticus* sp. n. is readily distinguished from all species of the genus *Dendronotus* by a light brownish ground colour with few distinct scattered white dots. There is little variation of colour in *Dendronotus arcticus* sp. n. compared to that of other *Dendronotus* species. The radula of *Dendronotus arcticus* sp. n. is similar to those of *Dendronotus albus* and *Dendronotus robilliardi* sp. n. but clearly differs by its pattern of central and lateral teeth. The radular differences include the presence of reduced furrows on the central teeth of *Dendronotus arcticus* sp. n.: *Dendronotus albus* and *Dendronotus robilliardi* sp. n. have no furrows on their central teeth, whereas the central teeth of the common North Atlantic species *Dendronotus frondosus* have deep furrows. The common North Atlantic and Arctic species *Dendronotus lacteus* differs considerably from *Dendronotus arcticus* sp. n. by its radula (central teeth with deep furrows), colour, and reproductive system. Other species of the genus *Dendronotus* clearly differ from *Dendronotus arcticus* sp. n. by radular patterns. The reproductive system of *Dendronotus arcticus* sp. n. differs from those of *Dendronotus albus* and *Dendronotus robilliardi* sp. n. by the presence of a twisted penis, by the colour pattern of the dorsal appendages, by the shape of the central tooth, and by the thicker vagina. *Dendronotus arcticus* sp. n. can be clearly distinguished from the recently described NW Pacific species *Dendronotus kamchaticus*, *Dendronotus kalikal*, and *Dendronotus primorjensis* by the colour and the radular and reproductive system patterns.

Uncorrected p-distances are different between *Dendronotus arcticus* sp. n. and the sympatric Arctic species *Dendronotus lacteus* (range 10.0–10.8 % for COI, and 1.6–1.8% for 16S data set), and *Dendronotus robustus* (range 12.8–13.9% for COI, and 3.2–3.4% for 16S). *P*-distances are different between *Dendronotus arcticus* sp. n. and the North Pacific *Dendronotus kamchaticus* (range 8.6–10.0% for COI, and 2.3–2.7% for 16S), *Dendronotus kalikal* (10.1 % for COI, and 2.3–2.5% for 16S), and *Dendronotus primorjensis* (range 12.0–12.5% for COI, and 2.5–2.7% for 16S). Minimum interspecific distances of the COI marker separate *Dendronotus arcticus* sp. n. from other species with high genetic divergence: 10.1% from *Dendronotus kalikal*,﻿ 9.3% from *Dendronotus kamchaticus*, 10.5% from *Dendronotus lacteus*, ﻿12.3% from *Dendronotus primorjensis*, and 13.4% from *Dendronotus robustus*.

#### Dendronotus robilliardi sp. n.

Taxon classificationAnimaliaNudibranchiaDendronotidae

http://zoobank.org/2BA57DC2-EFC9-4662-8A9C-931F69589DE9

Figs 2 , 3B

**Figure 2.**
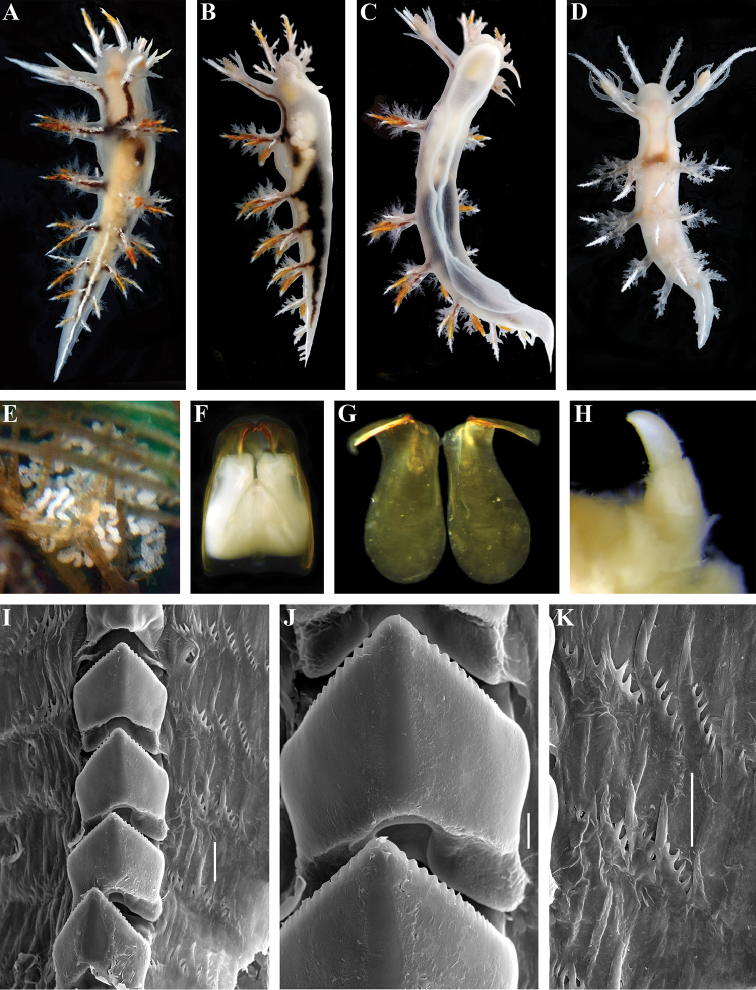
*Dendronotus robilliardi* sp. n.: **A** holotype ZMMU Op-568, live, dorsal view **B** same, lateral view **C** same, ventral view **D** paratype ZMMU Op-569, live, dorsal view **E** egg mass *in situ*, same collection data as holotype **F** paratype ZMMU Op-567, jaws and radula *in situ*, dorsal view **G** same, jaws, lateral views **H** same, penis **I** same, posterior rows of radula, SEM **J** same, details of central teeth, SEM **K** same, details of lateral teeth, SEM. Scale bars **I, K** = 30 µm **J** = 10 µm. Photos of living specimens by Nadezhda Sanamyan, other photos and SEM images by Alexander Martynov.

**Figure 3.**
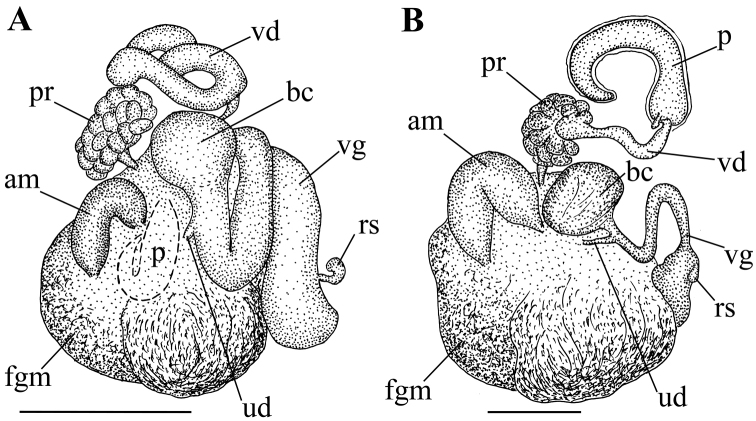
Reproductive systems: **A** *Dendronotus arcticus* sp. n., holotype ZMMU Op-561 **B** *Dendronotus robilliardi* sp. n., paratype ZMMU Op-567. Abbreviations: **am** ampulla; **bc** bursa; **fgm** female gland mass; **pr** prostate; **p** penis; **rs** receptaculum semenis; **u** uterine duct; **vd** vas deferens; **vg** vagina. Drawings by Tatiana Korshunova. Scale bars 1 mm.

**Figure 4.**
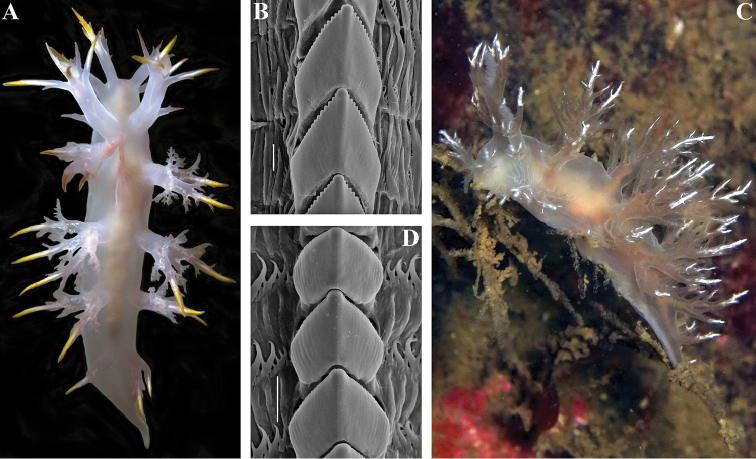
**A** *Dendronotus albus* MacFarland, 1966, live specimen ZMMU Op-566, dorsal view, Rich Passage, NE Pacific **B** same, posterior radular teeth, SEM **C** *Dendronotus kamchaticus* Ekimova et al., 2015, live specimen ZMMU Op-565, dorsal and lateral view, Rich Passage, NE Pacific **D** same, posterior radular teeth, SEM. Scale bars **B, D** = 30 µm. Photos of living specimens by Karin Fletcher, SEM images by Alexander Martynov.

Dendronotus albus : Robilliard 1970: 466–470, pl. 64, fig 34, text figs 2–4, 6, 22–24 (excluding part of Geographical section, p. 469–Baja California); Morris et al. 1980: 332–333, part; McDonald 1983: 172, part; Koh 2006: locality information and photo; Lloyd 2007: locality information and photo; Martynov et al. 2015b: 74–75 pp, figs 2в, г; Ekimova et al. prepublication: 2–10, figs 2D–F, 3D–G, 4A (non albus MacFarland, 1966).

##### Type material.

Holotype, ZMMU Op-568, 35 mm long (live), NW Pacific, Kamchatka, Starichkov Island, 52°47.009'N–158°36.185'E, 17.09.2015, depth 11.5 m, stones, SCUBA diving, collector N.P. Sanamyan. 1 paratype, ZMMU Op-567, same locality and collectors as holotype. 1 paratype, ZMMU Op-447, same locality and collectors as holotype. 1 paratype (egg mass only), ZMMU Op-570, same locality and collectors. 1 paratype, ZMMU Op-569, NW Pacific, Kamchatka, Zhirovaya Bay, 52°36.767'N–158°27.318'E, 12.06.2016, depth 18 m, stones, SCUBA diving, collector N.P. Sanamyan.

##### Type locality.

The NW Pacific, Kamchatka, Russia.

##### Etymology.

In honour of Gordon Robilliard (Gig Harbor, Washington State, USA), the author of the classic study on the genus *Dendronotus*, including the description of *Dendronotus diversicolor* Robilliard, 1970. For a long time Robilliard attempted to resolve status of *Dendronotus diversicolor* (Behrens 2006); *Dendronotus diversicolor* was finally synonymised with *Dendronotus albus* based on molecular data forty years later by Stout et al. (2010) (see also Discussion below). Here molecular evidence is provided showing the existence of another species in the NW Pacific belonging to the *Dendronotus albus* complex; therefore, this is a good opportunity to honour the important contributions of Gordon Robilliard to the systematics of the genus *Dendronotus*, and particularly to the *Dendronotus albus* species complex problem.

##### Diagnosis.

5–6 pairs branched dorsolateral appendages, digestive gland penetrates 3–4 pairs of dorsolateral appendages, general colour translucent white, dorsolateral appendages colour variable, orange-copper pigment present or completely lacking, tips opaque white, opaque white stripes on tips of dorsal appendages and tail, central tooth with up to 15 small distinct denticles without furrows, vas deferens short, conical penis.

##### Description.

Body elongate, 30–35 mm in length (Fig. 2A–D). 4–5 branched appendages of oral veil, 4–6 appendages of rhinophoral stalks, 11–12 rhinophoral lamellae, unbranched (or with few small branches) rhinophoral lateral papilla present, 5–6 pairs larger branched dorsolateral appendages and 1–3 pairs smaller unbranched appendages reaching tip of tail, 5–10 lip papillae. Dorsolateral appendages with moderate primary stalk and secondary branches, and pointed tertiary branches, digestive gland penetrates 3–4 pairs of dorsolateral appendages including posterior ones (Fig. 2A–D). Reproductive and anal openings placed laterally on right side.

General colour translucent white with opaque white stripes on oral veil appendages, rhinophoral sheaths, posterior part of dorsum and on tips of dorsal appendages; orange-copper marks in middle part of dorsal and oral processes (Fig. 2A–C), or absent (Fig. 2D).

Dorsal processes of jaws inclined posteriorly at approximately 60° to longitudinal axis of jaw body and 0.45 of its length (Fig. 2F–G). Masticatory borders with ridge-like denticles. Radula formula 43 × 3–9.1.9–3. Central tooth with up to 15 small distinct denticles (Fig. 2I, J), without furrows. Lateral teeth slightly curved, bearing up to seven distinct long denticles (Fig. 2K).

Reproductive system triaulic (Fig. 3B). Ampulla wide, folded twice. Prostate moderate in size, consists of *ca.* 19–20 alveolar glands. Vas deferens short, relatively narrow, penial sheath elongate, relatively long , curved, conical penis (Fig. 2H). Vagina narrow, bent, moderate in length, distally expanded into vestibulum. Uterine (insemination) duct short. Bursa copulatrix large, irregularly spherical, stalked, small oval seminal receptaculum placed distally on vestibulum (Fig. 3B).

##### Biology.

Inhabits stones and rocky bottom. Feeds on the hydroid *Abietinaria annulata* (Kirchenpauer, 1884).

##### Distribution.

The type specimens of *Dendronotus robilliardi* sp. n. originate from the NW Pacific, Kamchatka, Russia. According to the ceratal pattern, a specimen of *Dendronotus albus* recorded from cold waters of South Korea, 37°7'N, 129°E (Koh 2006) is also likely to be *Dendronotus robilliardi* sp. n.; therefore, a very broad distribution of *Dendronotus robilliardi* sp. n. is expected in the NW Pacific, from the Commander Islands in the north to Korea in the south. According to the morphological data given in Robilliard (1970) and a detailed image by Lloyd (2007) clearly showing up to six pairs of dorsolateral appendages (three of them contain digestive gland branches), the range of *Dendronotus robilliardi* sp. n. in NE Pacific reaches at least British Columbia and Washington State (San Juan Island). Robilliard and Barr (1974) also presented a record of *Dendronotus albus* from Alaska without an image. Since Robilliard consistently misidentified *Dendronotus albus* in his revision (1970) (see Discussion and Table 2), most probably the Alaskan record also belongs to the species *Dendronotus robilliardi* sp. n. However, the majority of the records *Dendronotus albus* from California and especially from Baja California (Robilliard 1970; Behrens 1980, 1991) probably represent true *Dendronotus albus*. The specimens of *Dendronotus albus* (= *Dendronotus robilliardi* sp. n.) which were studied by Robilliard (1970) originated from San Juan Island, Washington State and Albert Head, British Columbia, whereas more southern records were listed according to the information from James Lance only (Robilliard 1970: 469; Bertsch et al. 1972: 305). A selection of detailed images of several specimens of *Dendronotus albus* from the type locality of this species, Monterey Bay, California (McDonald 2016) showing only specimens with four to five pairs (the fifth pair if present is smaller) of dorsolateral appendages is in a full agreement with the first description of true *Dendronotus albus* (MacFarland, 1966). Sandra Millen (pers. comm.) has distinguished *Dendronotus albus* *sensu* Robilliard, 1970 from *Dendronotus diversicolor* (a synonym of *Dendronotus albus*, ﻿see Table 2) in the British Columbia region. *Dendronotus diversicolor* was also recorded without an illustration from British Columbia by Lambert (1976). A record of *Dendronotus diversicolor* by Millen (1989) from Alaska represents the northernmost range of true *Dendronotus albus* since that specimen had four ceratal pairs plus a small bump, and digestive gland extending in to the two anterior pairs (S. Millen, pers. comm.).

**Table 2.** Key diagnostic characters of *Dendronotus albus* MacFarland, 1966, its synonym *Dendronotus diversicolor* Robilliard, 1970, *Dendronotus albus* sensu Robilliard 1970 (= *Dendronotus robilliardi*), and *Dendronotus robilliardi* sp. n.

	*Dendronotus albus* (based on the original description, MacFarland 1966)	*Dendronotus* “*albus*” (from Robilliard 1970)	*Dendronotus diversicolor* (based on the original description, Robilliard 1970)	*Dendronotus diversicolor* (from Ekimova et al. prepublication)	*Dendronotus* “*albus*” (from Ekimova et al. prepublication)	*Dendronotus robilliardi* sp. n. (present study)
Locality	NE Pacific, California (type locality)	NE Pacific	NE Pacific, Washington (type locality)	NE Pacific	NW Pacific, Kamchatka and Kurile Islands	NW Pacific, Kamchatka (type locality)
Body length (live)	Up to 30 mm	Up to 40 mm	Up to 73 mm	Appr. 40 – 50 mm	Appr. 20 mm	Up to 35 mm
Number of pairs of dorsolateral appendages (“cerata”)	4–5	5–7 (4–8)	4–5	4–5	5–6	5–9
Digestive gland branches in dorsolateral appendages	Only in 2 anterior pairs	Up into 6 pairs, including posterior ones	Only in 2 anterior pairs	Only in 2 anterior pairs	In 4–5 pairs	In 3–4 pairs, including posterior ones
Colour of dorsolateral appendages	"With orange-yellow stripe becoming a dark-brown termination in a clear tip"	Variable, orange, copper, tip opaque white, both orange and white pigments may completely lacking	Variable, opaque orange or opaque white, including tips	“Yellow pigment on cerata only on the tips, pigment occurs in epidermal cells”	“Internal yellow pigment near the base; tips with white pigment”	Variable, orange-copper, tip opaque white, orange pigment may completely lacking
Jaws	-	The dorsal processes at 50–60° to the longitudinal axis, 0.43 × of its length	The dorsal processes at 60° to the longitudinal axis, and about 0.4 × of its length	-	-	The dorsal processes of the jaws at approximately 60° to the longitudinal axis, and 0.45 × of its length
Denticles of jaws	Ridge-like denticles (according to Pl. 47, Fig. 4–11)	Ridge-like denticles	-	-	-	Ridge-like denticles
Radula formula	36–38 × (7.9.1.7.9)	32–38 × (6.8.1.6–8)	33–38 × (6–9.1.6–9)	34 × 8.1.8	34–38 × 7–9.1.7–9	43 × 3–9.1. 9–3
Central teeth	16–20 denticles	11–14 (7–17) denticles	13–17 (7–25) denticles	10–17 denticles	10–17 denticles	Up to 15 denticles
Lateral teeth	5–7 denticles	4–6 (3–8) denticles	4–10 (2–14) denticles	4–10 denticles	4–10 denticles	up to 7 denticles
Ampulla	Wide, bent (according to Pl. 50, Fig. 4)	"Very wide, short, crescentic"	"Wide, which is folded against itself for most of its length"	"Well-developed, which is folded against itself for most of its length"	"Wide and short, crescent-shaped"	Wide, folded twice
Relative size of discoid prostate	Large (according to Pl. 50, Fig. 4)	"Much smaller than in *Dendronotus diversicolor*"	Large (according to Fig. 28)	"Quite large"	Small	Moderate
Number of prostatic alveolar glands	"some ten"	12–15	"30 or more"	10	10	19–20
Vas deferens	Short, widened after prostate, then narrowed (according to Pl. 50, Fig. 4)	"Relatively short, quite narrow"	Short, wide	Short, wide	Narrow	Short, relatively narrow
Penis	"Short, nearly straight, tapering to a blunt tip"	"Moderately long, narrow, tapered to a point"	"Short, wide, nearly straight, tapers gradually to a blunt tip"	Relatively straight, tapers gradually to a blunt tip (according to Fig. 4B)	Conical (according to Fig. 4A)	Relatively long, conical
Vagina	Narrow (according to Pl. 50, Fig. 4)	"Quite narrow"	Narrow	Relatively wide (according to Fig. 4B)	Narrow (according to Fig. 4A)	Narrow
Uterine (insemination) duct	Short (according to Pl. 50, Fig. 4)	Short	Short	Long	Short	Short
Bursa copulatrix	"Spherical, almost sessile"	"Spherical, stalked"	"Squashed ovoid", "stalked"	Spherical, non-stalked (according to Fig. 4B)	Spherical, non-stalked (according to Fig. 4A)	Irregularly spherical, stalked
Seminal receptaculum	"Small, pyriform"	"Long, flaccid, sac-like"	"Small, spherical"	Relatively small (according to Fig. 4B)	Relatively large (according to Fig. 4A)	Small, oval

Both *Dendronotus robilliardi* sp. n. and true *Dendronotus albus* evidently may co-occur in some localities around at least the British Columbia/Washington waters. *Dendronotus albus* was recently recorded and illustrated from the Salish Sea (Washington) by Fletcher (2013), geographically thus very close to San Juan Island, the type locality of *Dendronotus diversicolor*, and from where also *Dendronotus* “*albus*” (= *Dendronotus robilliardi* sp. n.) was already reported by Robilliard (1970). Thus *Dendronotus robilliardi* sp. n. appears to be a boreal species widely distributed in the northern Pacific and adapted for lower temperatures compared to *Dendronotus albus*. The latter species is mostly likely distributed in the NE Pacific from British Columbia southwards potentially to Baja California, in warmer temperature conditions.

##### Remarks.

**Figure 5.**
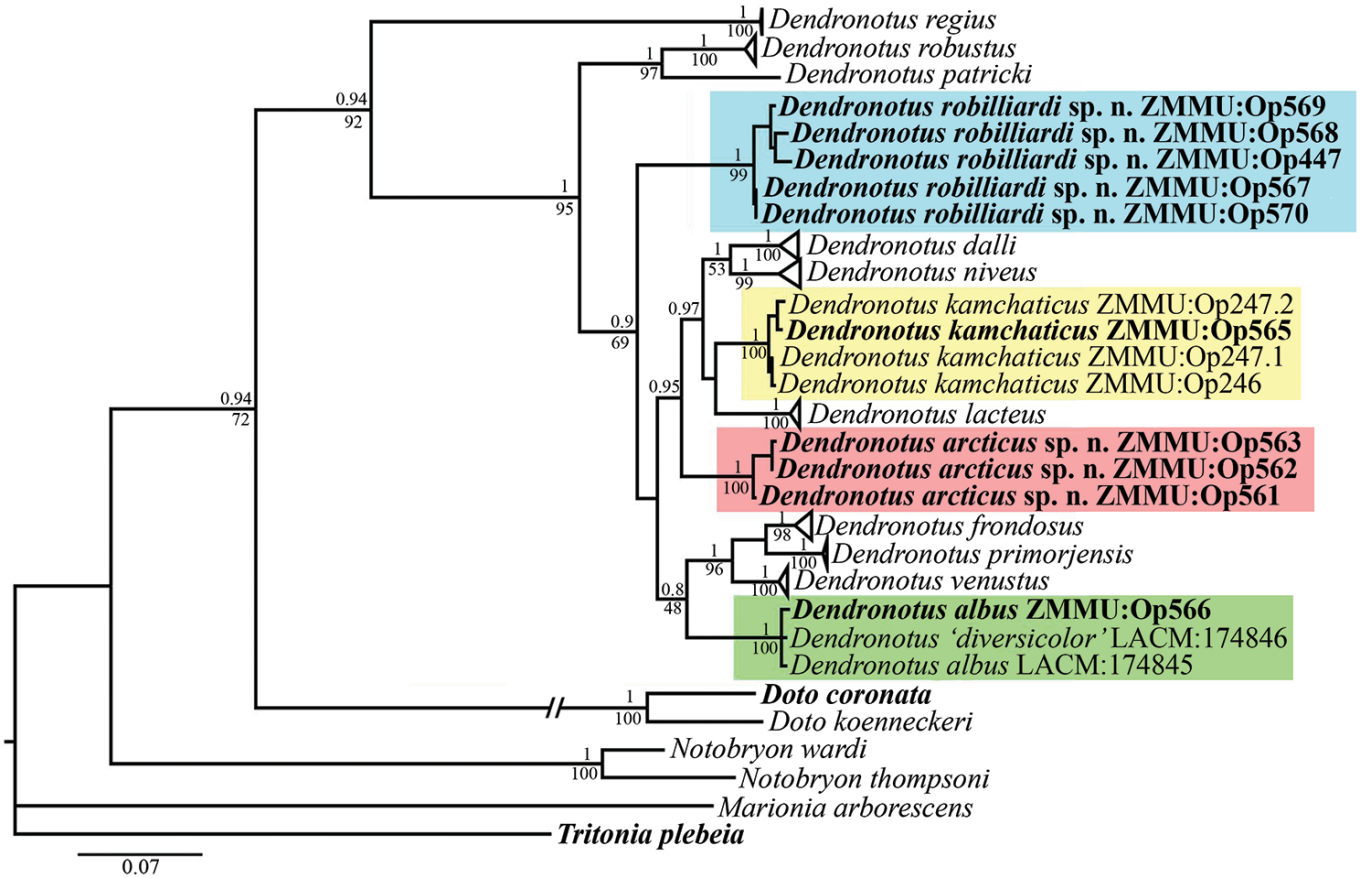
Phylogenetic tree based on combined molecular data (COI + 16S + 28S) represented by Bayesian Inference. Numbers above branches represent posterior probabilities from Bayesian Inference. Numbers below branches indicate bootstrap values for Maximum Likelihood. Some branches are collapsed at species level. New specimens are highlighted in bold.

There is a significant genetic gap between *Dendronotus robilliardi* sp. n. and the morphologically similar *Dendronotus albus* (13.6–14.5% for COI gene, 2.3–2.5% for 16S gene) (Fig. 5). According to Carmona et al. (2013) such values can be considered as species- and genus-‘level’ differences in the nudibranch molluscs. *Dendronotus robilliardi* sp. n. is also distinguished morphologically from the true *Dendronotus albus* MacFarland, 1966. *Dendronotus albus* (including its synonym *Dendronotus diversicolor*) has only 4–5 pairs of dorsolateral appendages and the digestive gland penetrates only the two anteriormost pairs of the dorsolateral appendages (see also Discussion). *Dendronotus robilliardi* sp. n. has 5–9 pairs of dorsolateral appendages and the digestive gland penetrates at least 3–4 pairs of the dorsolateral appendages. In his redescription of *Dendronotus albus* Robilliard (1970) misidentified this species, as did Ekimova et al. (prepublication): *Dendronotus albus* *sensu* Robilliard (1970) shares a larger number of pairs of dorsolateral appendages with *Dendronotus robilliardi* sp. n. and not with *Dendronotus albus* (= *Dendronotus diversicolor*) and can be referred to this new species. Table 2 outlines the differences between these two species.

*Dendronotus robilliardi* sp. n. differs both morphologically and according to the genetic distances from its sympatric species *Dendronotus dalli* (range 12.0–14.0% for COI, and 2.7–3.2 % for 16S), *Dendronotus kalikal* (range 10.8–12.1% for COI, and 3.2 - 3.4% for 16S), and *Dendronotus kamchaticus* (range 12.5–13.7% for COI, and 2.8–3.2% for 16S). Another NW Pacific species, *Dendronotus primorjensis*, also differs from *Dendronotus robilliardi* sp. n. by external morphology, radular and reproductive features, and by *p*-distances (range 12.2–13.6% for COI, and 3.2–3.7% for 16S). Minimum interspecific distances of the COI marker separate *Dendronotus robilliardi* sp. n. from other species with high genetic divergences: 13.0% from *Dendronotus dalli*, 11.5% from *Dendronotus kalikal*, 12.9% from *Dendronotus kamchaticus*, and 12.8% from *Dendronotus primorjensis*.

#### Dendronotus kamchaticus

Taxon classificationAnimaliaNudibranchiaDendronotidae

Ekimova et al., 2015

Dendronotus kamchaticus Ekimova, Korshunova, Schepetov, Neretina, Sanamyan & Martynov, 2015: 869–872, figs 6E, 8D, 16A, B, 17, 18A.

##### Material.

1 specimen, ZMMU Op-565, NE Pacific, Puget Sound, Rich Passage, Washington State, USA, 47 °58.7'N–122°54.65'W, 17.03.2014, depth 17.4 m, stones and algae, SCUBA diving, collector Karin Fletcher.

##### Description.

Body elongate, 30 mm in length (live specimen, Fig. 4C). Four branched appendages of oral veil, *ca.* five appendages of rhinophoral stalks, approximately ten rhinophoral lamellae, branched rhinophoral lateral papilla present, six pairs dorsolateral appendages, *ca.* 10–15 lip papillae. Dorsolateral appendages with long primary stalk and secondary branches, and elongate tertiary branches (Fig. 4C). Reproductive and anal openings placed laterally on right side. General colour pale, translucent white with few scattered brown dots and opaque white stripe on dorsal appendages (Fig. 4C).

Dorsal processes of jaws inclined posteriorly at approximately 70° to longitudinal axis of jaw body and 0.37 of its length. Masticatory borders with fine denticles. Radula formula 44 × 3–10.1.10–3. Central tooth with reduced or completely absent denticles and furrows in posterior rows (Fig. 4D); anteriormost juvenile rows denticulated. Lateral teeth short, slightly curved, bearing up to six distinct denticles.

Reproductive system triaulic. Ampulla wide, folded twice. Prostate consists of approximately 20–25 alveolar glands. Vas deferens relatively short and expands to oval penial sheath and conical penis. Vagina moderate in length. Bursa copulatrix large, rounded, elongated, with small seminal receptaculum placed distally.

##### Biology.

Inhabits stony and rocky substrates.

##### Distribution.

According to the present data *Dendronotus kamchaticus* has a broad transpacific distribution in the northern part of the Pacific Ocean.

##### Remarks.

*Dendronotus kamchaticus* was recently described from Kamchatka in the Russian NW Pacific (Ekimova et al. 2015). Here an outstanding and unexpected record of *Dendronotus kamchaticus* is presented from the American NE Pacific (Washington state). The single collected specimen matches closely with *Dendronotus kamchaticus* from the type locality, and genetic distances between the four *Dendronotus kamchaticus*,﻿﻿ including *Dendronotus kamchaticus* from Washington waters, range from 0–1.1% for COI, and 0–0.2% for 16S. Mean *p*-distance value of the COI marker within *Dendronotus kamchaticus* group is 0.7%. Furthermore, *Dendronotus kamchaticus* from Washington waters share an important diagnostic character with those from the type locality, the central teeth with strongly reduced denticles and furrows in the posterior radular teeth (Fig. 4D) and denticulated anteriormost juvenile rows. This is the first record of *Dendronotus kamchaticus* from the NE Pacific, approximately 6000 km away from the type locality, across the ocean.

## Discussion

*Dendronotus arcticus* sp. n. is the first species of the genus described from the central Arctic region of Eurasia. Using a combination of external and internal morphological characters, *Dendronotus arcticus* sp. n. can be distinguished from all recently reviewed species of the genus *Dendronotus* (Stout et al. 2010, Ekimova et al. 2015, Martynov et al. 2015a, b). The molecular data also support the description of *Dendronotus arcticus* sp. n. as a new species.

*Dendronotus albus* species complex is a long standing problem of the North Pacific nudibranch taxonomy. Since Robilliard (1970) described the species *Dendronotus diversicolor* there was little consensus on how to distinguish this species from *Dendronotus albus*. Recently, using morphological and molecular data, *Dendronotus diversicolor* was considered to be junior synonym of *Dendronotus albus* (Stout et al. 2010). While this paper was under review, a manuscript appeared online (Ekimova et al. prepublication): these authors recognised the presence of two species and suggested that *Dendronotus albus* inhabits the NW Pacific and that *Dendronotus diversicolor* is a separate NE Pacific species, challenging the previous synonymy by Stout et al. (2010). However, there are number of key problems with their assumptions:

1. Ekimova et al. prepublication called a species from NW Pacific “true” *Dendronotus albus* but this is in error since the first description of *Dendronotus albus* in MacFarland (1966) was based on specimens from NE Pacific (California) and fits well with the diagnostic features of *Dendronotus diversicolor* (type locality also in NE Pacific, Washington) and was previously synonymised by Stout et al. (2010).
2. According to the original description in Robilliard (1970: 471) *Dendronotus diversicolor* possesses four to five pairs dorsolateral appendages (cerata), and the digestive gland penetrates only the two anteriormost pairs of the cerata; precisely these characters have been reported in the original description of *Dendronotus albus* (MacFarland 1966: 275, 278–279) but were not noted by Ekimova et al. (prepublication). These facts also support the synonymy of *Dendronotus diversicolor* with *Dendronotus albus*.
3. Colour patterns of *Dendronotus albus* and *Dendronotus diversicolor* vary greatly (Robilliard 1970) and cannot serve as reliable diagnostic features; in Ekimova et al. (prepublication) only specimens with yellow pigment are discussed whereas white specimens without yellow/orange pigment are common (Robilliard 1970).
4. Ekimova et al. (prepublication) recorded the body length as approximately 20 mm as a diagnostic feature for “true *Dendronotus albus*”, while for *Dendronotus diversicolor* they recorded 40–50 mm; however, this is inaccurate since MacFarland (1966: 276) in the original description of *Dendronotus albus* recorded 30 mm, and Robilliard (1970: 466) reported the length up to 40 mm, which clearly overlaps with the size of *Dendronotus diversicolor*. The notion “true” is thus incorrect, since the species reported by Robilliard and in the publication of Ekimova et al. (prepublication) is in fact *Dendronotus robilliardi* sp. n. and not *Dendronotus albus*.
5. The number of prostatic alveolar glands cannot be diagnostic as pointed out by Ekimova et al. (prepublication) because there is too much variation within species; while they reported no more than ten alveoli for NW Pacific *Dendronotus albus* (= *Dendronotus robilliardi* sp. n.), the present work records no less than 19–20 alveoli in *Dendronotus robilliardi* sp. n. from the same NW Pacific region while Robilliard (1970: 473) also reported no less than 30 alveoli in the original description of *Dendronotus diversicolor*. For *Dendronotus albus* MacFarland (1966: 279) reported only “some ten” prostatic alveoli, but the prostate itself is large (MacFarland 1966, Plate 50, Fig. 4) and similar to the original description of *Dendronotus diversicolor* (Robilliard 1970, fig. 28) and not to the small prostate of *Dendronotus albus* *sensu* Robilliard (1970, fig. 24 = *Dendronotus robilliardi*). Thus, the number of prostatic alveolar glands should be used for diagnostic purposes with great care since their number may depend on the physiological condition of a specimen, and also because it is very easy to make a mistake during counting of the alveoli under a stereomicroscope.
6. The same considerable variation can be mentioned for other reproductive features. E.g. the uterine (insemination) duct of *Dendronotus diversicolor* is short according to the original description (Robilliard 1970: 473–474) whereas according to Ekimova et al. (prepublication) it is long, but these authors claim that “these features were described by Robilliard (1970) for *Dendronotus diversicolor* as important for its separation from *Dendronotus albus*”. Furthermore, the bursa copulatrix (termed as receptaculum in Ekimova et al. (prepublication) although Stout et al. 2011 provided an updated nomenclature), the receptaculum semenis (termed bursa in Ekimova et al. prepublication), and the shape of the ampulla are all variable and variably described by authors (Table 2). Thus, Robilliard’s (1970) and Ekimova`s et al. (prepublication) statements that reproductive characters are important for distinguishing of *Dendronotus albus* and *Dendronotus diversicolor* should be reconsidered.

Thus, *Dendronotus albus* (according to the original description) is essentially similar to *Dendronotus diversicolor*, but differs considerably from *Dendronotus robilliardi*. All sequenced specimens of *Dendronotus albus* species complex from the NE Pacific (including the present study) show distinct species-level molecular differences compared to NW Pacific *Dendronotus robilliardi*. In this study, a very large specimen (70 mm long) of *Dendronotus albus* from the NE Pacific (Washington State, Rich Passage, 17 March 2014, 12.5 m, collector Karin Fletcher, ZMMU Op-566) (Fig. 4A, B) was studied: it had four pairs dorsal appendages, and thus morphologically matches true *Dendronotus albus* and the original description of its synonym *Dendronotus diversicolor*. In our phylogenetic analysis (Fig. 5) the *Dendronotus albus* specimen from Rich Passage is robustly placed in the same clade with the other *Dendronotus albus* (including those named *Dendronotus diversicolor*), and all four specimens of *Dendronotus robilliardi* sp. n. and its egg mass (Fig. 2E) are clustered in a single separate clade (Fig. 5). The number of radular rows in our 70 mm long *Dendronotus albus* is 38, somewhat less than the 43 rows in half as small specimen of *Dendronotus robilliardi*. The number of prostatic lobules in *Dendronotus albus* from Rich Passage is *ca.* 25-27, thus approaching the range reported for *Dendronotus diversicolor* by Robilliard (1970) and supports the size-dependence theory of the number of prostatic alveoli.

This work confirms the synonymy of *Dendronotus diversicolor* Robilliard, 1970 as a junior synonym of *Dendronotus albus* MacFarland, 1966 as suggested by Behrens (2006) and realized by Stout et al. (2010), and the existence of a third species described herein as *Dendronotus robilliardi* sp. n. (Table 2). Further work on more material is desirable to confirm the actual range of this species in the northeastern Pacific.

*Dendronotus kamchaticus* was described recently (Ekimova et al. 2015) and was thought to be endemic of the NW Pacific. However, in this study a surprising record of *Dendronotus kamchaticus* from NE Pacific is documented. The specimen from the Puget Sound, Rich Passage, agrees well with *Dendronotus kamchaticus* from the NW Pacific in radular patterns and molecular data but differs in having a much paler ground colour and the presence of dense white pigment (Fig. 4C). The pale ground colour is similar to that of another species, *Dendronotus dalli*, and to the pale variants of *Dendronotus venustus*. Misidentifications with *Dendronotus dalli* and *Dendronotus venustus* may explain the absence of records of *Dendronotus kamchaticus* from the NE Pacific, and records of these two species need to be re-examined in light of this study. Other explanations may include anthropogenic transportation by ships, either in the biofouling organisms or as larvae in ballast tanks; however, *Dendronotus kamchaticus* may prove to be a species with a natural transpacific distribution. We also observe differences from the original description in Ekimova et al. (2015), including the shape of the dorsal processes (long slender branches of the dorsal processes not short bulbous ones described by Ekimova et al. 2015) and patterns of the masticatory processes of jaws. Therefore, the diagnosis of *Dendronotus kamchaticus* is expanded to include denticles on the masticatory processes of the jaws and elongated dorsal processes.

## Conclusions

In this study new data on the taxonomy, phylogeny, and biogeography of the genus *Dendronotus* are presented. A true Arctic species *Dendronotus arcticus* sp. n. from the central Eurasian coastal zone is described. This species is well supported by both morphological and molecular data. A long-standing problem of *Dendronotus albus* species complex is revisited and for the first time it is clearly concluded that Robilliard (1970) in the course of his revision of the genus *Dendronotus* misidentified true *Dendronotus albus* as it was originally described by MacFarland (1966) (Table 2). The key diagnostic characters of *Dendronotus albus* fully agree with the original description of *Dendronotus diversicolor* Robilliard, 1970, and the latter is confirmed a junior synonym of *Dendronotus albus*. At the same time, a species that was redescribed by Robilliard (1970) under the name *Dendronotus albus* has considerable differences from the true *Dendronotus albus* but is the newly described *Dendronotus robilliardi* from the NW Pacific. Finally, a remarkable record of *Dendronotus kamchaticus* is presented here for the first time from NE Pacific, extending its range to the east by some 6000 km.

## Supplementary Material

XML Treatment for Dendronotus arcticus

XML Treatment for Dendronotus robilliardi

XML Treatment for Dendronotus kamchaticus
